# Physicochemical and bioactive constituents, microbial counts, and color components of spray-dried *Syzygium cumini* L. pulp powder stored in different packaging materials under two controlled environmental conditions

**DOI:** 10.3389/fnut.2023.1258884

**Published:** 2023-10-04

**Authors:** Vishal Kumar, Chandra Shekhar Singh, Shiva Bakshi, Sudhir Kumar, Satya Prakash Yadav, Zakarya Ali Saleh Al-Zamani, Pankaj Kumar, Upendra Singh, Kamlesh Kumar Meena, Durga Shankar Bunkar, Vinod Kumar Paswan

**Affiliations:** ^1^Department of Dairy Science and Food Technology, Institute of Agricultural Sciences, Banaras Hindu University, Varanasi, India; ^2^Department of Food Technology, School of Life Sciences and Biotechnology, CSJMU, Kanpur, India; ^3^Department of Food Technology & Science, Faculty of Agriculture and Veterinary Medicine, Ibb University, Ibb, Yemen; ^4^Department of Agriculture Engineering, SKN College of Agriculture, SKNAU, Jobner, Rajasthan, India; ^5^Department of Dairy and Food Microbiology, College of Dairy and Food Technology, MPUAT, Udaipur, India

**Keywords:** anthocyanins, controlled environments, accelerated environment, packaging materials, powder properties, storage conditions, total phenolics

## Abstract

Currently, the demand for functional food items that impart health benefits has been rising. Blackberry (*Syzygium cumini* L.) fruit has high anthocyanin content and other functional attributes. However, this seasonal fruit is highly perishable, and a large proportion of it goes unharvested and wasted worldwide. Spray drying of the fruit pulp can impart improved shelf life, ensuring long-term availability for consumers to exploit its health benefits. The storage quality varies according to the type of packaging material and the storage environment. Therefore, in this study, the shelf life span of the spray-dried *Syzygium cumini L*. pulp powder (SSCPP) was investigated during 6 months of storage under three types of packaging materials (i.e., polystyrene, metalized polyester, and 4-ply laminates) in a low-temperature environmental (LTE) and at ambient environmental conditions. The physicochemical stability of bioactive principles (TPC and TAC), microbial counts, and color components were analyzed at 0, 2, 4, and 6 months of storage. There was a significant gradual loss of dispersibility and solubility with an increase in flowability, bulk density, and wettability during the entire storage period for all three packaging materials. The TSS, pH, TPC, TAC, and microbial counts decreased in the SSCPP both at ambient and LTE conditions during the study. Among all the packaging materials, the 4-ply laminate was found to be the most appropriate and safe for storage of spray-dried SCPP at LTE conditions.

## 1. Introduction

Over the past few decades, the interest of consumers in natural food items consisting of bioactive principles and functional attributes has accelerated tremendously because of their divergent health benefits (1). *Syzygium cumini* L., generally called jambolan, Indian blackberry, black plum, Portuguese plum, and Malabar plum across the world, is such a fruit that encompasses multitudinous beneficial phytochemicals, antioxidants, phenolic compounds, and other nutrients. This fruit is single-seeded, elliptical, succulent, and fleshy, berry-type, along with a purple mesocarp and a blackish-purple pericarp (2). Furthermore, these fruits are incredibly perishable; hence, they cannot be transported over a long distance for marketing or export as fresh produce, generating a postharvest loss of well over 6.7–15.88% in India (3). *Syzygium cumini* L. fruits have been reported to contain numerous pharmacological activities, including scavenging of free radicals, anticarcinogenic, hepatoprotective, hypoglycemic, anti-diarrheal, and antidiabetic characteristics (4–7). *Syzygium cumini* L. fruit polyphenols have been demonstrated to display strong antioxidant and antimicrobial properties against some infectious microorganisms such as *Staphylococcus aureus, Aeromonas hydrophila, Klebsiella pneumoniae, Escherichia coli*, and *Candida albicans*. The pulp of this fruit also possesses anti-quorum sensing and antibiofilm properties, aiding in its antimicrobial properties (8–10). Anthocyanins, the water-soluble flavonoid compounds present in the *Syzygium cumini* L. fruit pulp, are also reported to show potent anticarcinogenic attributes such as apoptosis and inhibition of tumor formation and its growth in animals (4, 11). Due to the nutritional, functional, medicinal, and delicious significance of *Syzygium cumini* L. fruit, several modern processing methods have been explored to make commercially acceptable products with extended shelf life and retained nutritional values. Numerous products, such as ready-to-serve health drinks, preserves, jam, leather, vinegar, squashes, jellies, and wines, have been prepared from *Syzygium cumini* L. fruits (7). The development of dried *Syzygium cumini* pulp powder (SCPP) has also been explored through freeze-drying (12), forced convection warm air drying (13), microwave hot air drying (14), foam mat drying (15), combined vacuum pulsed osmotic dehydration (16), spouted bed drying (17), and spray drying (18). Furthermore, when it comes to heat-sensitive ingredients, spray drying is an appropriate technique and has been widely used in the production of commercially dried powders of vegetables and fruits. The main advantages involve reduced water activity, better reconstitution properties, and easy transportation and storage (19).

The method of preparation and processing, storage temperature, kind of packaging material used to protect the product, and packaging environment are known to influence the longevity of the powder. The selection of proper packaging materials with optimum barrier properties is of prime importance for lengthening the shelf life of the fruit powder by preserving volatile flavors and color characteristics and retaining antioxidants and other bioactive compounds (20, 21). Fruit powders are generally packaged in heat-sealed multi- or monolayers of aluminum laminates, for example, polyethylene laminated with aluminum foil (22). To sustain the fruit powders, all such parameters are kept in mind to maintain proper nutritive value along the storage length. In their study, Pereira et al. (23) reported that spray-dried jucara pulp could safely be packed in polyethylene/metalized polyester packaging for a storage period of up to 103 days. Similarly, packaging tomato powder in metalized polyester films was more effective as compared to low-density polyethylene against deteriorative quality changes during the 6-month storage period (24). Recently, Ravindran et al. (25) demonstrated that banana inflorescence retained its quality characteristics when packaged in metalized polyethylene-polyester pouches throughout the 2 months of storage at ambient temperature without the formation of any peroxides. Similarly, aluminum-laminated polyethylene pouches are better suited for packaging spray-dried papaya powder at 38°C and 90% relative humidity (RH) (26). Although SCPP has been developed by several workers employing different modern drying and dehydration techniques, the storage study of SCPP is still awaited. Furthermore, storage temperature and humidity are critical variables in the packaging and may directly influence the stability of phenolics, anthocyanins, and other bioactive components (27, 28). Therefore, the experimentation was planned to scientifically explore and investigate the ultimate effect of packaging materials and storage environments on the retention of physicochemical, bioactive compound stability, microbiological, and functional attributes of spray-dried SCPP (SSCPP). The stored SSCPP was analyzed every 2 months (0, 2, 4, and 6 months) of storage intervals.

## 2. Materials and methods

### 2.1. Raw materials

The acquisition of freshly ripened *Syzygium cumini* L. fruits was done from the market close to Varanasi, India (25.3119° N, 83.0120° E) during the period of early monsoon (i.e., from mid-June to mid of July). The Maltrin 500^®^ RM 1249 (maltodextrin) and other chemicals used in this research were procured from HiMedia Pvt. Ltd., Mumbai, India. Packaging materials, such as polystyrene (PS; 10 μm), metalized polyester (MPEST; 12 μm), and 4-ply laminates (4-ply LAM; 12 μm), were supplied by the Indian Institute of Packaging, New Delhi, India.

### 2.2. Preparation of SSCPP

SSCPP was prepared from *Syzygium cumini* L. fruits as per Singh et al. (18) using a pilot-scale spray dryer. Briefly, *Syzygium cumini* L. pulp was blended with an appropriate proportion (600 ml/kg of *Syzygium cumini* L. pulp) of water and maltodextrin (10% of the pulp) and stirred well for approximately 30 min until homogeneity was achieved. The homogenized *Syzygium cumini* L. pulp slurry was then fed into a pilot-scale spray dryer (PRODUCTION MINOR™ Spray Dryer, GEA Process Engineering China Limited, Shanghai, China). The feed rate, air pressure, and inlet temperature of drying air were standardized at 18–20 rpm, 4 kg/cm^2^, and 185°C, respectively. The completion of the process was achieved as the air inlet and outlet temperatures fell relatively below 100°C and 75°C. Subsequently, the samples were attained from a product collection vessel.

### 2.3. Packaging and storage of SCPP

The SCPP (50 g) was packed in 15 cm × 8 cm pouches of PS, MPEST, and 4-ply LAM, and all pouches were carefully sealed using a vacuum sealer (Vac Master SVP-50, Japan). After packaging and sealing, all the pouches were stored in an ambient (30°C and RH 75%) and low-temperature environment (LTE) (5°C and RH 59%). LTE was maintained by placing a saturated magnesium nitrate solution in a desiccator placed in a low-temperature incubator (model: LTI-700, made by Eyela, Japan). Similarly, an RH of 75% was maintained by placing a saturated solution of sodium chloride in the desiccator inside a BOD chamber for recreating the ambient environment. These storage temperatures and humidity conditions were selected based on the practical applicability and suitability of storing the powder at normal household fridge temperature or at ambient temperature, which was also supported by earlier studies based on different spray-dried powders (26, 29, 30).

### 2.4. Physicochemical analysis of SCPP

The physical properties of SCPP such as moisture content (AOAC; 930.15) (31), water activity (aw) (by water activity meter; Aqua Lab Pre, Decagon Devices, United States), solubility, wettability, flowability, dispersibility, and bulk density (32) and the chemical properties such as pH (by pH meter; LAB India Instruments Pvt. Ltd., India), titratable acidity (TA; % citric acid equivalent), and total soluble solids (TSS) (33) were determined as per the standard procedures.

### 2.5. Total phenolic content

The determination of TPC was according to the Folin Ciocalteau (FC) method illustrated by Koh et al. (34) with slight modifications. The sample in methanol solution was prepared by dissolving 10 mg of SSCPP in 30 ml of methanol, followed by centrifugation at 6,000 *g* for 10 min. The supernatant obtained after extraction was filtered using filter paper (Whatman No. 4). Subsequently, 2.5 ml of the 0.2 N FC reagent was added to the extract (0.5 ml) and allowed to react for 5 min. Then, 2 ml of sodium carbonate (75 g/L) was added to the reaction mixture and diluted to 25 ml using distilled water. Finally, the resultant mix was incubated for 2 h at room temperature, and the absorbance was recorded at 760 nm by using a UV–Vis spectrophotometer **(**V-1800, Shimadzu Corporation, Kyoto, Japan**)**, where methanol was used as a blank. The standard calibration curve was generated using tannic acid (0–100 mg/L). The TPC value was mentioned in terms of mg tannic acid equivalents (TAE/g) of the SCPP.

### 2.6. Total anthocyanin content

The analysis of TAC was performed using a pH differential method as described by Pandey et al. (35). Two serial dilutions of SSCPP were made for each of the developing stages, one with a 0.025 M buffer solution of potassium chloride at pH 1.0 and the other with a 0.4 M buffer solution of sodium acetate at pH 4.5, respectively. This was obtained by adopting a predetermined dilution factor and then the dilutions were equilibrated for 15 min at room temperature. Absorbances were obtained at 700 and 510 nm against distilled water as a blank using a UV–Vis spectrophotometer. The concentration of monomeric anthocyanin pigment was expressed as cyanidin-3-glucoside (Sigma Aldrich Chemicals Pvt. Ltd., Bengaluru, India), being the largest anthocyanin component present in SSCPP. The calculation of TAC was carried out by using the ensuing formula:

$$\begin{array}{l} TAC\;\left(mg\;L^{-1}\right)=\left(\frac{\text{Ab}\times \text{MW}\times \text{DIF}\times 100}{e}\right)\;1 \end{array}$$

where *Ab* = [(*Ab*_510 *nm*_− *Ab*_700 *nm*_) *pH* 1.0 ]−[(*Ab*_510 *nm*_− *Ab*_700 *nm*_) *pH* 4.5]

Ab = absorbance; MW = molar weight of cyanidin-3-glucoside, i.e., 449.2; DIF = dilution factor; cell path length (l) = 1 cm; molar absorptivity (€) = 26,900.

### 2.7. Color estimation

The color property of spray-dried SSCPP was estimated with the help of a Lovibond Spectrocolorimeter (Model: PFXi880/F, Lovibond House, Sun Rise Way, Amesbury, Salisbury, SP4 7GR, United Kingdom). The CIE (International Commission on Illumination) L^*^ (light/dark), b^*^ (yellow/blue), and a^*^ (red/green) values were observed. The calculation of chroma (C^*^) with respect to color intensity and hue angle (H°) was done using the below-mentioned formula. Furthermore, the hue angles of 0°, 90°, 180°, and 270° depict pure red, yellow, green, and blue colors, respectively. The hue angle represents the perception of color, whereas chroma portrays the saturation of color (36).

$$\begin{array}{l} {\text{C}}^{{}^{*}}=\sqrt{\left({{\text{a}}^{*}}^{2}\;+{{\text{b}}^{*}}^{2}\right)}\;\\ {H\;=\tan}^{-1}\left(\frac{b^{{}^{*}}}{a^{{}^{*}}}\right)\; \end{array}$$

### 2.8. Microbiological counts of SSCPP

The microbial counts adjudicating the total plate, spore (37), and rapid yeast and mold count (RYM) (AOAC; 2014.05) (38) of the SSCPP during the period of storage were performed. An amount of 0.1 g of SSCPP was added to normal saline (10 ml), and subsequently serial dilutions were prepared. The Standard Plate Count (SPC) was carried out using plate count agar (HiMedia Pvt. Ltd., Mumbai, India), followed by the pour plate method and consequent incubation at 37°C for approximately 72 h. For determining RYM, potato dextrose agar was used. After the inoculation of plates, incubation was carried out at 37°C for a time period of 48 h. The calculation of spore count was done in tryptone glucose yeast agar, with incubation of inoculated plates for 48 h at 55°C. The microbial colonies formed were thereafter counted, and the obtained results were presented in terms of colony-forming units (CFU)/g of the samples.

### 2.9. Statistical analysis

The resultant data for each parameter were collected in triplicate. The data were analyzed statistically by ANOVA using the IBM SPSS Statistics for Windows, version 20 (IBM Corp., Armonk, NY, United States). The first factor was packaging materials, and the second factor was the storage periods. Tukey's test was used to evaluate the difference in means. The data were expressed as mean ± SD (standard deviation) along with the critical difference (CD), which is the least significant difference; above this, all the differences were declared significant (at a 95% confidence level).

## 3. Results

### 3.1. Powder properties of the spray-dried SCPP

The process for formulation of spray-dried SCPP had been optimized earlier with 185°C inlet air temperature, 10% maltodextrin (w/v), and maximal TPC and TAC retention. This optimized spray-dried SCPP was analyzed for physicochemical, microbial, and powder properties and sensory evaluation, and then the powder was used for the shelf-life investigation during 6 months of storage. Physicochemical characteristics and bioactive principles (TPC and TAC) in the freshly prepared SCPP were analyzed and are presented in Table 1. The moisture content, TA, bulk density, TAC, and TPC of the freshly prepared SSCPP at 0 days of storage were 2.98%, 0.63%, 0.41 g/ml, 102.5 mg/100 g, and 202.1 g GAE/100 g, respectively. These values were close to the values reported earlier for freshly prepared SCPP by the spray drying method (18, 39). The TPC and TAC values were in accordance with Shelke et al. (40) who also concluded that gum arabica and maltodextrin were more effective for producing SSCPP with appropriate functional, physical, color, and flow attributes. Second, in comparison to *Syzygium cumini* L. fruit pulp prepared by Santos et al. (9), the moisture content and TA after spray drying were reduced from 83.51 to 2.98% and 0.65 to 0.63%, respectively, while the TSS was increased from 12.93 to 31.43°Brix. The decrease in moisture content values observed in the present study was in agreement with the results obtained by Ferrari et al. (41) for spray-dried blackberry powder. Moreover, in the spray-drying process, maltodextrin is responsible for enhancing the solid content, which in turn leads to a higher TSS of the end products (42). Besides, for better yield and physicochemical properties, the clarification of pulp by using Pectinase enzyme (0.09% enzyme concentration) at 33°C for 75 min has been recommended (43).

**Table 1:** Physicochemical properties and bioactive components of fresh *Syzygium cumini L*. pulp powder before storage.

**Attributes**	**Contents**
Moisture (%)	2.98 ± 0.05
Water activity (a_w_)	0.23 ± 0.02
TSS (°Brix)	31.43 ± 0.4
Titratable acidity (% citric acid)	0.63 ± 0.2
Flowability (q)	48.67 ± 1.47
Wettability (s)	89.23 ± 1.28
Solubility (%)	98.01 ± 1.82
Bulk density (g/ml)	0.44 ± 0.2
Dispersibility (%)	91.43 ± 1.66
TAC (mg/100 g)	100.71 ± 0.02
TPC (g GAE/100 g)	201.12 ± 0.04

Values are presented as mean ± standard deviation (*n* = 3); TAC, total anthocyanins; TPC, total phenolics.

### 3.2. Physical properties of SSCPP during storage

**Figure 1:**
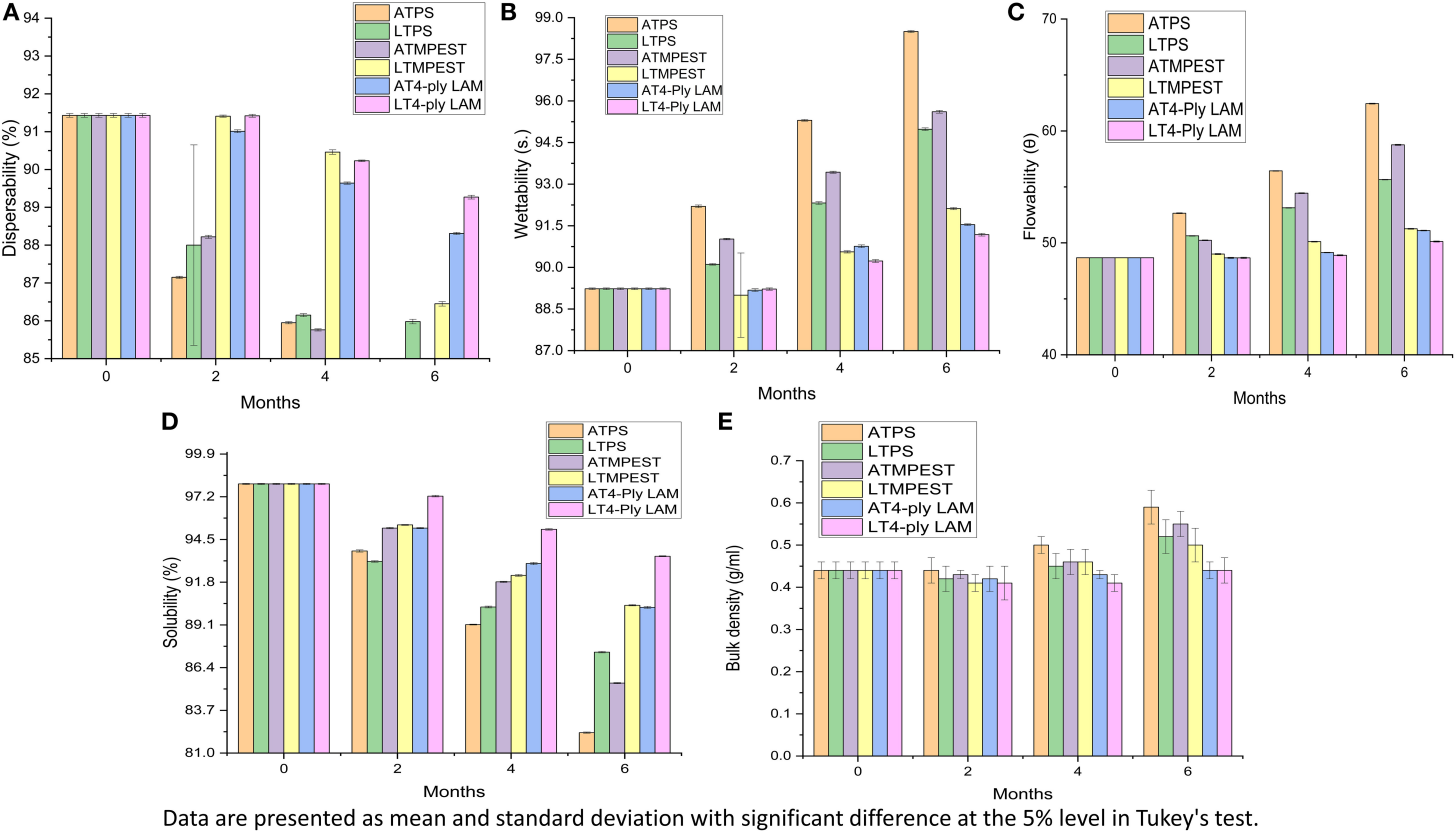
Changes in physical properties of *Syzygium cumini L*. pulp powder during storage. **(A)** Dispersibility, **(B)** wettability, **(C)** flowability, **(D)** solubility, and **(E)** bulk density. ATPS, ambient temperature polystyrene; LTPS, low-temperature polystyrene; ATMPEST, ambient temperature metalized polyester; LTMPEST, low-temperature metalized polyester; AT4-ply LAM, ambient temperature 4-Ply laminates; LT4-ply LAM, low-temperature 4-Ply laminates.

The packaging material impact on the physical attributes of SSCPP stored in ambient and low-temperature environments has been presented in Figure 1. Indeed, there was a gradual loss in dispersibility and solubility; instead, bulk density, flowability, and wettability increased with storage. When compared to storage at ambient temperature, the losses in the physical quality of SSCPP were less at lower temperatures. Furthermore, the losses pertaining to flowability, bulk density, wettability, dispersibility, and solubility were faster for POL-packed samples when compared to MPEST and 4-ply LAM. Alternatively, the deprivation in physical attributes of SSCPP was lowest in 4-ply LAM stored at LTE in comparison to the other combinations of packaging and storage environment.

Flowability, wettability, and bulk density are important parameters to be evaluated while handling, bag filling, transportation, and storage of powdered products (44). The flowability property of the powder fell with increasing order of storage interval at elevated temperatures and RH. Faster loss in dispersibility, solubility, flowability, and wettability of the SCPP packed in POL may be attributed to its higher permeability and water vapor transmission rate (WVTR) in comparison to MPEST and 4-ply LAM (45). Similar to the present study, an increase in bulk density of spray-dried *Annona muricata* L. powder stored for 91 days has also been reported by Chang et al. (46). The attributed escalation in bulk density might be due to an increase in van der Waals forces among the powder particles as they stay closer in the packed environment, making them more compact (47). Furthermore, an increment in the quantity of free fatty acids (reflected by increased TA) on storage leading to a rise in bulk density may also be contributing to the loss in wettability and flowability. Similar to the present findings, Chauhan and Patil (29) also expressed that this bulk density increase causes poorer flowability in the mango powder when stored at 21°C than that of the powder kept at 10°C. For spray-dried spinach powders, an increment in wettability was displayed during the 1^st^ month, with a non-significant decrease until the 4^th^ month of storage (48). They also found significantly higher wettability at elevated temperatures, which leads to the development of cake-like structure and stickiness, which might prevent subsequent wetting more readily. Nevertheless, as a limitation of the present study, variations in moisture content and water activity over the storage period contributing to the changes in these physicochemical attributes have not been investigated.

#### 3.2.1. pH and titratable acidity

The pH and TA of SSCPP stored under different environmental conditions are presented in Table 2. During the entire storage period, a gradual reduction in pH value was seen. For all types of packaging, the pH diminished approximately from 3.12 to 2.84 in the ambient environment and from 3.12 to nearly 2.60 at LTE during the 6-month storage period. The decrease in pH at the end of 6 months of storage was greater (*P* < 0.05) in PS when compared to MPEST and 4-Ply LAM. The decreased pH was reflected by the significant increase in TA content (Table 3). Although statistically significant, a qualitatively minor increase in the TA was observed regardless of the packaging type. This implies an interplay between various constituents, ultimately leading to chemical changes (49). Similar to the present study, a time-dependent pH reduction of guava pulp powder stored in polyethylene packages was reported by Breda et al. (50) under both the ambient environment (at 25°C and 75% RH) and the accelerated conditions (at 35°C and 90% RH). Other observations where the pH tended to fall during the storage period were also pointed out in mandarin powder by Kadam et al. (51), in cherry and plum tomato powder by Obadina et al. (52), and in sweet corn kernel powder by Kumar et al. (53). The decreased pH value prevents non-enzymatic browning (54) and thus might contribute in maintaining the color stability of the stored SSCPP. Moreover, Korese et al. (55) reported that for retaining the pH value of *Gardenia erubescens* fruit powder, the high-density polyethylene (HDPE) package was most suitable when compared with the low-density polyethylene (LDPE), polyethylene (PE), and paperboard.

**Table 2:** Chemical and bioactive constituents of spray-dried *Syzygium cumini L*. pulp powder stored in different packaging materials in ambient (30°C and RH 75%) and low-temperature (5°C and RH 59%) environmental conditions.

**Packaging materials**	**Storage conditions**	**Storage months**	**pH**	**Total soluble solids (°Brix)**	**Titratable acidity (%)**	**TPC (g GAE/100 g)**	**TAC (mg/100 g)**
Polystyrene	Ambient temperature	0	3.12 ± 0.04	33.45 ± 0.03	0.55 ± 0.01	201.12 ± 0.04	100.71 ± 0.02
		2	3.05 ± 0.03^b^	32.50 ± 0.06^c^	0.59 ± 0.04^b^	188.01 ± 0.01^c^	98.14 ± 0.04^c^
		4	2.98 ± 0.04^b^	31.52 ± 0.05^b^	0.64 ± 0.01^a^	180.22 ± 0.03^b^	94.21 ± 0.05^b^
		6	2.34 ± 0.02^a^	30.67 ± 0.03^a^	0.72 ± 0.03^a^	174.98 ± 0.04^a^	84.50 ± 0.10^a^
	Low temperature	2	3.02 ± 0.02^b^	33.42 ± 0.05^c^	0.56 ± 0.02^a^	198.21 ± 0.04^c^	99.32 ± 0.05^c^
		4	2.78 ± 0.03^ab^	32.50 ± 0.53^b^	0.59 ± 0.03^a^	187.26 ± 0.04^b^	96.24 ± 0.03^b^
		6	2.60 ± 0.26^a^	31.43 ± 0.04^a^	0.60 ± 0.02^a^	184.65 ± 0.04^a^	89.21 ± 0.03^a^
Metalized polyester	Ambient temperature	2	3.10 ± 0.20^b^	33.01 ± 0.03^c^	0.57 ± 0.01^a^	190.13 ± 0.03^c^	98.56 ± 0.04^c^
		4	2.91 ± 0.08^b^	32.78 ± 0.03^b^	0.62 ± 0.05^ab^	185.65 ± 0.03^b^	93.21 ± 0.04^b^
		6	2.45 ± 0.05^a^	31.40 ± 0.03^a^	0.69 ± 0.03^b^	180.43 ± 0.02^a^	85.49 ± 0.04^a^
	Low temperature	2	3.00 ± 0.26^a^	33.44 ± 0.03^c^	0.56 ± 0.06^a^	197.54 ± 0.03^c^	99.53 ± 0.06^c^
		4	2.87 ± 0.04^a^	33.22 ± 0.04^b^	0.57 ± 0.06^a^	188.43 ± 0.03^b^	97.75 ± 0.06^b^
		6	2.64 ± 0.03^a^	31.01 ± 0.04^a^	0.60 ± 0.03^a^	186.48 ± 0.09^a^	88.76 ± 0.04^a^
4-Ply laminates	Ambient temperature	2	3.11 ± 0.03^b^	33.44 ± 0.06^c^	0.56 ± 0.04^a^	190.86 ± 0.04^c^	100.20 ± 0.03^c^
		4	3.03 ± 0.03^b^	33.12 ± 0.04^b^	0.58 ± 0.01^a^	187.54 ± 0.03^b^	99.32 ± 0.04^b^
		6	2.96 ± 0.05^a^	32.41 ± 0.03^a^	0.60 ± 0.03^a^	185.32 ± 0.09^a^	90.17 ± 0.07^a^
	Low temperature	2	3.12 ± 0.02^b^	33.43 ± 0.04^b^	0.55 ± 0.03^a^	200.14 ± 0.04^c^	100.50 ± 0.05^c^
		4	3.01 ± 0.04^a^	33.41 ± 0.04^b^	0.56 ± 0.03^a^	198.75 ± 0.05^b^	99.76 ± 0.05^b^
		6	2.98 ± 0.05^a^	33.14 ± 0.04^a^	0.58 ± 0.04^a^	195.72 ± 0.05^a^	95.14 ± 0.03^a^

Data are presented as mean ± standard deviation (*n* = 3) and means forerun by different lowercase letters in the same column within the same storage period; results were significantly different at the 5% level in Tukey's test.

**Table 3:** Color components of spray-dried *Syzygium cumini L*. pulp powder stored in different packaging materials in ambient (30°C and RH 75%) and low-temperature (5°C and RH 59%) environmental conditions.

**Packaging materials**	**Storage conditions**	**Storage months**	L^*^value	a^*^value	b^*^value	**Chroma**	**Hue angle**
Polystyrene	Ambient temperature	0	76.92 ± 0.02	30.72 ± 0.02	−18.56 ± 0.02	28.20 ± 0.02	−29.03 ± 0.03
		2	78.23 ± 0.02^c^	32.09 ± 0.03^c^	−15.71 ± 0.54^c^	26.02 ± 0.02^c^	−27.09 ± 0.06^c^
		4	81.31 ± 0.04^b^	35.32 ± 0.02^b^	−13.21 ± 0.03^b^	23.09 ± 0.02^b^	−21.02 ± 0.05^b^
		6	85.03 ± 0.02^a^	38.21 ± 0.02^a^	−10.34 ± 0.06^a^	18.03 ± 0.02^a^	−17.85 ± 0.03^a^
	Low temperature	2	76.99 ± 0.04^c^	31.22 ± 0.03^c^	−17.34 ± 0.03^c^	26.98 ± 0.01^c^	−28.32 ± 0.03^c^
		4	77.22 ± 0.02^b^	33.37 ± 0.01^b^	−15.32 ± 0.05^b^	24.42 ± 0.02^b^	−23.07 ± 0.01^b^
		6	78.51 ± 0.03^a^	36.04 ± 0.03^a^	−12.54 ± 0.02^a^	20.54 ± 0.06^a^	−19.05 ± 0.06^a^
Metalized polyester	Ambient temperature	2	76.98 ± 0.04^c^	31.01 ± 0.03^c^	−17.00 ± 0.02^c^	27.02 ± 0.02^c^	−27.76 ± 0.02^c^
		4	78.32 ± 0.01^b^	33.44 ± 0.02^b^	−15.29 ± 0.04^b^	24.01 ± 0.04^b^	−24.76 ± 0.03^b^
		6	82.04 ± 0.02^a^	35.34 ± 0.03^a^	−12.55 ± 0.02^a^	20.04 ± 0.07^a^	−20.08 ± 0.01^a^
	Low temperature	2	76.43 ± 0.03^c^	30.02 ± 0.01^c^	−17.86 ± 0.06^c^	27.76 ± 0.04^c^	−28.53 ± 0.02^c^
		4	76.62 ± 0.02^b^	32.03 ± 0.03^b^	−16.07 ± 0.01^b^	25.53 ± 0.05^b^	−26.07 ± 0.02^b^
		6	77.32 ± 0.04^a^	34.24 ± 0.04^a^	−13.65 ± 0.04^a^	21.65 ± 0.04^a^	−23.53 ± 0.02^a^
4-Ply laminates	Ambient temperature	2	77.04 ± 0.01^c^	30.89 ± 0.04^b^	−18.06 ± 0.03^c^	27.96 ± 0.04^c^	−28.73 ± 0.02^c^
		4	77.82 ± 0.03^b^	32.30 ± 0.57^b^	−17.18 ± 0.04^b^	26.28 ± 0.04^b^	−26.34 ± 0.03^b^
		6	79.21 ± 0.05^a^	32.70 ± 0.59^a^	−15.97 ± 0.04^a^	23.64 ± 0.05^a^	−26.62 ± 0.05^a^
	Low temperature	2	76.90 ± 0.03^a^	30.83 ± 0.03^c^	−18.44 ± 0.06^c^	28.06 ± 0.14^c^	−28.96 ± 0.04^c^
		4	77.28 ± 0.57^a^	31.23 ± 0.02^b^	−17.98 ± 0.07^b^	27.43 ± 0.04^b^	−27.65 ± 0.02^b^
		6	77.03 ± 0.02^a^	32.22 ± 0.04^a^	−16.75 ± 0.02^a^	25.03 ± 0.02^a^	−25.71 ± 0.55^a^

Data are presented as mean ± standard deviation (*n* = 3) and means forerun by different lowercase letters in the same column within the same storage period; results were significantly different at the 5% level in Tukey's test.

#### 3.2.2. Total soluble solids

In all the treatments, TSS content decreased over the storage period, and at the end, the values reached were statistically different from the initial ones for all evaluated samples (Table 3). Nonetheless, the TSS decline in the 6-month storage in an ambient environment was greater (*P* < 0.05) in PS (from 33.45 to 30.67%) in comparison with MPEST (from 33.45 to 31.40%) and 4-Ply LAM (from 33.45 to 32.41%). The decrease in the TSS of SSCPP was in agreement with the findings of earlier work carried out by Costa et al. (56). The reason for this might be the breakdown of solids during storage. This reduction in TSS may be attributed to a lowering of nutritional characteristics. The effect of storage on volatile fatty acids and their degradation/oxidation also depends on the permeability of the packaging material. Furthermore, Ankush et al. (57) also reported a decrease in TSS of solar-dried wild *ber* fruit on storage. However, Adetoro et al. (58) reported that TSS of hot air-dried pomegranate aril increased during storage, which might be due to the caramelization reaction occurring at a higher temperature. Yet, as reported by Farooq et al., the TSS of freeze-dried tomato powders was higher than that of hot-air-dried samples (59). Alongside, Verma et al. also reported a reduction in TSS of mango powder on day 90 when stored in aluminum pouches (60).

### 3.3. Total phenolics and anthocyanins

Both the TPC and TAC values of SSCPP diminished with storage due to the oxidation of these components. Although the decrease in TPC and TAC was noticeable only after the 2nd month of storage in the case of all packaging materials, it increased during the later period of storage, and the reduction was greater during 4–6 months of storage. Furthermore, the reduction in TPC and TAC was greater in the ambient environment as compared to LTE, i.e., higher retention of anthocyanin in spray-dried bayberry powder in the course of 6 months of storage at low temperature (4°C) as compared to ambient temperature. The acceleration in degradation of anthocyanin at elevated temperatures is generally related to the Maillard reaction, which takes place in the presence of proteins and reducing sugars during a time span of longer storage and is intensified with the presence of oxygen (61). The 4-Ply LAM and MPEST have significantly (*P* < 0.05) improved retention of TPC and TAC in contrast to PS packaging material. The least reduction in TPC and TAC was observed in SCPP stored in 4-Ply LAM at LTE, even after 6 months, in comparison to other packaging materials used in this study. The reduction in TPC at LTE was from 201.12 to 195.72 mg GAE per 100 g of SSCPP, whereas the TAC was reduced from 100.71 to 95.14 mg per 100 g in 4-Ply LAM. Our findings are in concurrence with Zoric et al. (22), who outlined that the gradual loss of phenolics in spray-dried sour cherry juice packed in PET/PPMET/PE or PET/AL/PE laminates was greater at 37°C than at 4°C. They further reported that anthocyanins degraded more readily as compared to other phenolics during their 91-day storage study. However, for spinach juice that is spray-dried, the degradation of phenolics almost remained stable after 2 months of storage (62).

Moreover, the loss of TPC and TAC at lower temperatures was less when compared to ambient storage temperatures. This might be due to the higher oxidation rates of phenolics at higher temperatures (63). Mishra et al. (64) also delineated the same results in terms of TPC loss of spray-dried hog powder. However, an escalating trend of TPC loss was observed in cantaloupe powder when kept in accelerated storage conditions (at approximately 38°C, 90% RH) and could possibly be related to microbial growth, reactions among the oxidized phenolics, and the formation of newer antioxidants with storage (65). Alongside, higher retention of TPC and TAC in 4-Ply LAM and MPEST in our study may be attributed to their ability to provide better barrier properties for oxygen transfer as compared to PS packaging material. Even though laminates do not significantly affect the stability of phenolics, storage temperature and length have the most pronounced effect on bioactive content (22).

### 3.4. Color components of SCPP during storage

The color components of the powder products derived from fruits and vegetables directly influence product quality and consumer acceptability. Hence, it is of foremost importance to examine the color profile of the resultant product. Even for powdered products, the brighter the color, the more likely it will be accepted (66). Anthocyanins (cyanidin, petunidin, and malvidin glucosides) present in *Syzygium cumini* fruit pulp may be responsible for the bright purple color of the SSCPP (67). The impact of different packaging materials on the color components of SSCPP is depicted in Table 3. Statistical analyses (ANOVA) depicted that the values of color components were affected significantly (*P* < 0.05) during the entire storage period stored at ambient (30°C and RH 75%) and LTE (5°C and RH 59%) conditions. During the storage period, the values of *L*, a^*^ were increased, whereas the b^*^ value, chroma, and hue angles decreased among all the packaging materials stored at ambient (30°C and RH 75%) and LTE (5°C and RH 59%) conditions, showing the improvement in redness and reduction in brightness. Moreover, the results demonstrated the drastic change in *L*, a^*^, and b^*^ for the PS packaging, whereas 4-ply laminate performed well under both environments by showing lesser variation during the entire storage period of 6 months. The MPEST also presented changes in all the color parameters, with a^*^ and b^*^ being the most affected ones. This can be well elucidated by the oxygen and water permeability of each respective packaging material. Nevertheless, studies have inferred that color change is generally correlated with factors such as water activity, moisture content, storage time and temperature, and sugar content, which causes non-enzymatic browning (65, 68). Likewise, Yian and Phing (69) also reported the same findings for the a^*^ and b^*^ values of kuini powder. In addition, Shishir et al. (30) demonstrated an increased trend in *L* values during the storage of pink guava powder.

The losses of color components were found more at ambient than in the low-temperature environment. Oxidation of anthocyanin may be responsible for the constant loss of color components of SSCPP stored in ambient and low-temperature environments. Muzaffar and Kumar (67) reported that the change in color profile of spray-dried tamarind pulp powder was degraded minimally when packed in glass; however, the loss of color values was significantly higher for the powder packed in LDPE. These changes may have occurred due to the Maillard reaction. Similarly, a steady reduction in the color value of mango milk power was also observed faster in polystyrene than in metalized polyester, 4-ply laminates, and tin cans (29). They also proved that a greater reduction was observed at 30°C than when stored at 5°C.

### 3.5. Microbiological counts of SSCPP during storage

The microbial enumeration of the SSCPP during storage was accomplished by evaluating the SPC, RYM, and spore count in the final product for the safety of consumers. The microbial counts in freshly prepared SSCPP (SPC, × 10^3^; RYM, × 10^1^; and spore counts, × 10^1^) were about 15.0, 6.4, and 10.3 CFU per g of the product, respectively (Table 4). The initial load of microbial counts mainly depends on the raw material quality and handling procedure of the product in preparation, processing, and packaging. The types of packaging material and temperature had a significant influence on the survival and growth of microbes in SSCPP. During the storage time, SPC, RYM, and spore count were in a downward trend in both the ambient environment and LTE conditions. In the ambient environment, the SPC, RYM, and spore count (log CFU per g of SSCPP) varied from 15.0 to 6.3, 6.4 to 2.1, and 10.3 to 7.4, respectively, during the entire storage period, while the SPC, RYM, and spore count (log CFU per g of SSCPP) at LTE varied from 15.0 to 7.6, 6.4 to 2.5, and 10.3 to 9.5, respectively.

**Table 4:** Microbial counts of spray-dried *Syzygium cumini L*. pulp powder stored in different packaging materials in ambient (30°C and RH 75%) and low-temperature (5°C and RH 59%) environmental conditions.

**Packaging materials**	**Storage conditions**	**Storage months**	**CFU × 10^3^**	**RYM × 10^1^**	**Spore count × 10^1^**
Polystyrene	Ambient temperature	0	15.00 ± 0.20	6.40 ± 0.10	10.30 ± 0.17
		2	13.20 ± 0.10^c^	5.27 ± 0.15^c^	9.80 ± 0.26^b^
		4	9.70 ± 0.53^b^	4.10 ± 0.46^b^	7.50 ± 0.20^a^
		6	6.80 ± 0.10^a^	1.80 ± 0.10^a^	7.10 ± 0.26^a^
	Low temperature	2	14.17 ± 0.12^c^	5.50 ± 0.36^c^	9.10 ± 0.26^b^
		4	10.70 ± 0.26^b^	4.20 ± 0.26^b^	8.50 ± 0.17^a^
		6	7.60 ± 0.17^a^	2.70 ± 0.17^a^	8.00 ± 0.20^a^
Metalized polyester	Ambient temperature	2	13.60 ± 0.26^c^	5.20 ± 0.17^c^	9.40 ± 0.44^b^
		4	10.80 ± 0.53^b^	4.40 ± 0.26^b^	8.60 ± 0.26^b^
		6	6.50 ± 0.26^a^	2.00 ± 0.26^a^	6.30 ± 0.26^a^
	Low temperature	2	14.30 ± 0.26^c^	5.70 ± 0.26^b^	9.40 ± 0.26^a^
		4	12.40 ± 0.10^b^	5.10 ± 0.53^b^	8.80 ± 0.53^a^
		6	9.50 ± 0.17^a^	3.20 ± 0.44^a^	8.60 ± 0.26^a^
4-Ply laminates	Ambient temperature	2	13.80 ± 0.26^c^	4.50 ± 0.26^c^	9.50 ± 0.20^c^
		4	10.20 ± 0.26^b^	3.30 ± 0.26^b^	8.70 ± 0.26^b^
		6	6.30 ± 0.26^a^	2.13 ± 0.59^a^	7.40 ± 0.10^a^
	Low temperature	2	14.70 ± 0.30^c^	6.20 ± 0.36^c^	10.20 ± 0.36^b^
		4	13.33 ± 0.21^b^	5.53 ± 0.15^b^	9.80 ± 0.10^ab^
		6	10.60 ± 0.17^a^	4.00 ± 0.10^a^	9.50 ± 0.26^a^

Data are presented as mean ± standard deviation (*n* = 3) and means forerun by different lowercase letters in the same column within the same storage period; results were significantly different at the 5% level in Tukey's test.

However, the decrease in microbial counts was more rapid at ambient conditions as compared to LTE during the entire duration of storage, irrespective of the packaging materials. Similar declining trends in microbial counts were also observed in convective dried mango milk powders during the storage study (29). The storage in MPEST depicted the lowest microbial count, and this finding was in accordance with Selvamuthukumaran and Khanum (70). The low water activity of the SSCPP and the antibacterial nature of some of the phytochemicals and polyphenols present in it, coupled with some antimicrobial chemicals unleashed in several degradation reactions, might have spawned fleet destruction of the microorganism's cells amid storage. Additionally, residual moisture is an important criterion, along with pH, which describes the level of microbial growth (71). Furthermore, the permeability of different constituents in the packages plays a crucial role in maintaining nutritional values and preventing microbial spoilage (72). *Syzygium cumini* L. fruit polyphenols and the synergism between multiple polyphenolic compounds present in *Syzygium cumini* L. fruits have been delineated to possess strong and broad-spectrum antimicrobial traits against some infectious microorganisms (8, 9, 73). As the action of phytochemicals on microorganisms and the chemical reaction rate are less at low temperatures, the reduction in microbes' number was established to be slower at LTE. Furthermore, the lowest microbial counts were observed in PS, followed by MPEST packaging at LTE. This might be due to the stiff, brittle nature of PS (74). Moreover, our results were also in accordance with the study published by Zaidi et al. (75), who reported a reduction in bacterial and yeast load after 6 months of storage at 4°C.

## 4. Conclusion

The selection of suitable packaging material and optimum storage environment conditions are critical factors for ensuring a longer shelf life of food products. In this study, the extended shelf life of SSCPP was achieved without the addition of any preservative. Among all packaging materials, the 4-ply LAM retained the better powder properties and bioactive profiles of SSCPP under LTE. Second, the PS was least suitable for packaging the spray-dried powder. In terms of microbial spoilage, the MPEST performed best, followed by the 4-Ply LAM. Overall, it can be concluded that 4-ply LAM can perform better for maintaining the nutritive and physicochemical profiles of SSCPP.

## Data availability statement

The original contributions presented in the study are included in the article/supplementary material, further inquiries can be directed to the corresponding author.

## Author contributions

VK: Conceptualization, Investigation, Visualization, Writing—original draft. CS: Conceptualization, Investigation, Methodology, Writing—original draft. SB: Data curation, Visualization, Writing—review and editing. SK: Data curation, Writing—review and editing. SY: Investigation, Writing—original draft. ZA-Z: Visualization, Writing—original draft. PK: Visualization, Writing—review and editing. US: Methodology, Writing—review and editing. KM: Writing—review and editing, Data curation. DB: Investigation, Methodology, Supervision, Writing—review and editing. VP: Conceptualization, Data curation, Supervision, Writing—original draft.
